# Extending Causality Tests with Genetic Instruments: An Integration of Mendelian Randomization with the Classical Twin Design

**DOI:** 10.1007/s10519-018-9904-4

**Published:** 2018-06-07

**Authors:** Camelia C. Minică, Conor V. Dolan, Dorret I. Boomsma, Eco de Geus, Michael C. Neale

**Affiliations:** 1 Department of Biological Psychology, Vrije Universiteit Amsterdam, Transitorium 2B03, Van der Boechorststraat 1, 1081 BT Amsterdam, The Netherlands; 2 Virginia Institute for Psychiatric and Behavioral Genetics, Virginia Commonwealth University, 1-156, P.O. Box 980126, Richmond, VA 23298-0126 USA

**Keywords:** Causality, Pleiotropy, Twin design, Mendelian randomization

## Abstract

Although experimental studies are regarded as the method of choice for determining causal influences, these are not always practical or ethical to answer vital questions in health and social research (e.g., one cannot assign individuals to a “childhood trauma condition” in studying the causal effects of childhood trauma on depression). Key to solving such questions are observational studies. Mendelian Randomization (MR) is an influential method to establish causality in observational studies. MR uses genetic variants to test causal relationships between exposures/risk factors and outcomes such as physical or mental health. Yet, individual genetic variants have small effects, and so, when used as instrumental variables, render MR liable to weak instrument bias. Polygenic scores have the advantage of larger effects, but may be characterized by horizontal pleiotropy, which violates a central assumption of MR. We developed *the MR-DoC twin model* by integrating MR with the Direction of Causation twin model. This model allows us to test pleiotropy directly. We considered the issue of parameter identification, and given identification, we conducted extensive power calculations. MR-DoC allows one to test causal hypotheses and to obtain unbiased estimates of the causal effect given pleiotropic instruments, while controlling for genetic and environmental influences common to the outcome and exposure. Furthermore, the approach allows one to employ strong instrumental variables in the form of polygenic scores, guarding against weak instrument bias, and increasing the power to detect causal effects of exposures on potential outcomes. Beside allowing to test pleiotropy directly, incorporating in MR data collected from relatives provide additional within-family data that resolve additional assumptions like random mating, the absence of the gene-environment interaction/covariance, no dyadic effects. Our approach will enhance and extend MR’s range of applications, and increase the value of the large cohorts collected at twin/family registries as they correctly detect causation and estimate effect sizes even in the presence of pleiotropy.

**Electronic supplementary material:**

The online version of this article (10.1007/s10519-018-9904-4) contains supplementary material, which is available to authorized users.

## Introduction

Establishing causality in observational studies is important as knowledge of the relationship between a putative causal factor (exposure) and a potential outcome may inform rational treatment and prevention policies. While randomized controlled trials (RCTs) are the acid test of causality, they are expensive, time consuming, and may be practically or ethically unfeasible. For example, one cannot assign randomly individuals to a ‘childhood trauma condition’ in studying the causal effects of’childhood trauma’ on’depression’. An important alternative to the RCT is Mendelian Randomization (Davey Smith and Ebrahim 2003).

Mendelian Randomization (MR) offers some traction in addressing causality by using genetic variants as instrumental variables to detect the causal effect of a modifiable risk factor (exposure) on a disease outcome in non-experimental settings (Evans and Davey Smith 2015; Davey-Smith and Hemani 2014). MR is quickly becoming one of the dominant approaches to establishing causality; many recent applications have been published (Evans and Davey Smith 2015; Ference et al. 2012, 2015; Vimaleswaran et al. 2013; Holmes et al. 2014a, b, c; Proitsi et al. 2014). The ascendency of MR is due to: dramatic drop in DNA genotyping costs, which has given rise to large samples of genotyped individuals, robust genetic associations established in genome-wide association studies (GWASs) (Welter et al. 2014), and the inherent advantages of MR, which include ecologic validity, robustness to reverse causation (from exposure to instrument) (Davey-Smith 2011) and confounding (Burgess and Thompson 2015).

MR requires instruments with a relatively strong association with the exposure. A disadvantage of many genetic variants is that they have weak effects (Visscher et al. 2012). Weak instruments confer insufficient statistical power, and render MR liable to weak instrument bias (Burgess and Thompson 2015; Davies et al. 2015; Burgess et al. 2011). Combining the weak genetic effects into a polygenic risk score (PGS) is a possible route to increase the strength of the genetic instrument (Burgess and Thompson 2013, 2015; Palmer et al. 2012; Pierce et al. 2010). However, the MR assumption that the instrument is not pleiotropic (has no direct effect on the outcome) is stronger in the case of a PGS instrument (Burgess and Thompson 2015; Burgess et al. 2011, 2014; Sheehan et al. 2008). A PGS comprises many genetic variants, any of which may directly affect both the exposure and the outcome, or may include variants in linkage disequilibrium with variants affecting the outcome. As demonstrated by twin studies (de Geus 2006; Kendler et al. 1992; Ligthart and Boomsma 2012; Middeldorp et al. 2005; Neale and Kendler 1995) and by polygenic risk score analyses (Evans et al. 2009; Purcell et al. 2009; Ligthart et al. 2014), many genetic variants associate with multiple phenotypes, suggesting pervasive pleiotropy (Sivakumaran et al. 2011; Bulik-Sullivan et al. 2015; Solovieff et al. 2013; Pickrell et al. 2016; Visscher and Yang 2016; Evans et al. 2013).

Several methods are currently in use as means to tackle the ‘no pleiotropy’ assumption [see also e.g., (Burgess et al. 2017; van Kippersluis and Rietveld 2017)]. Some approaches apply prior selection criteria to increase the probability that the instruments are valid. For instance, the stepwise procedure implemented in the R-package gtx (Johnson and Johnson 2012) employs iteratively a heterogeneity test to discard from a polygenic score genetic instruments yielding significant heterogeneity in the estimates of the causal effect. Possible heterogeneity is assumed to be indicative of pleiotropy. The efficiency of this method depends on its power to detect heterogeneity arising from pleiotropy. However, heterogeneity may arise from sources other than pleiotropy, so that one may needlessly weaken the instrument by removing valid genetic variants. Stepwise heterogeneity tests for identifying pleiotropy may suffer also from over-fitting, and may become difficult to interpret in the presence of many pleiotropic instruments. As acknowledged by the authors, the performance of this procedure in terms of bias and type I error rates within the MR context has yet to be established. Other approaches like those based on the median estimator (Bowden et al. 2016) can handle information from up to 50% invalid instruments. However, the strong MR assumption still applies to the variant(s) yielding the median causal effect. MR-Egger regression (Bowden et al. 2015) is another approach that, with weaker assumptions, gives consistent estimates even when all instruments are pleiotropic. The estimator is (asymptotically) consistent (i.e., the estimate of the causal effect converges to the true value with increasing sample size) under the assumption that the effect of the instrument on the exposure is uncorrelated with the direct effect of the instrument on the outcome (i.e., the Instrument Strength Independent of Direct Effect assumption). As the authors note, this assumption may not hold universally (Bowden et al. 2015). Furthermore, Bowden et al. noted (Bowden et al. 2015) that there are other plausible paths from the instrument to the outcome (in addition to the direct path, or the indirect path, via the exposure). For example, a possible path is via confounders affecting both traits, or due to linkage disequilibrium between the instrument and a genetic variant affecting the outcome. In this case the estimate of the estimates of the causal effect in Egger regression will likely be biased (Bowden and Jackson 2015). Finally, although MR-Egger uses multiple genetic variants to estimate the causal effect, these instruments are employed individually (i.e., not combined in a polygenic score). Strong instruments in the form of polygenic scores are desirable, not only to ensure sufficient statistical power, but also to preclude weak instrument bias.

The utility of the classical twin design (CTD) in the study of direction of causality is well established (De Moor et al. 2008; Duffy and Martin 1994; Heath et al. 1993; Kohler et al. 2011; Turkheimer and Harden 2000). The present aim is to combine MR and CTD into a single model. A similar approach was proposed by Kohler et al. who integrated CTD with the instrumental variable method (Kohler et al. 2011). We focus on the issues of identification and statistical power associated specifically with a (poly-)genic instrument, which may be related directly to the outcome (i.e., violating the ‘no-pleiotropy’ assumption). Integrating MR with CTD has three advantages: (1) it allows one to relax the strong MR assumption concerning the instrument’s conditional independence of the outcome (conditional on the exposure and confounders, i.e., the no pleiotropy assumption, also known as the exclusion restriction assumption; (2) by accounting for pleiotropy, the approach facilitates the use of PGS as instruments; and (3) in specific circumstances, the approach confers substantial gains in power relative to the standard MR approaches.

## Methods

Direction of Causation (DoC) twin model was advanced as an exploratory approach to establish direction of causation between two correlated traits (Heath et al. 1993; Gillespie et al. 2003; Verhulst et al. 2012; Verhulst and Estabrook 2012). In contrast, MR (Smith and Hemani 2014; Burgess and Thompson 2015) is used to test unidirectional causation (from designated causal exposure to outcome).^1^ Here we propose the MR-DoC twin model, developed by imposing restrictions on the DoC parameters to represent unidirectional causality hypotheses, and by extending the model to include measured genetic variants as instrumental variables. Integrating MR with DoC allows us to test certain MR assumptions.

**Fig. 1:**
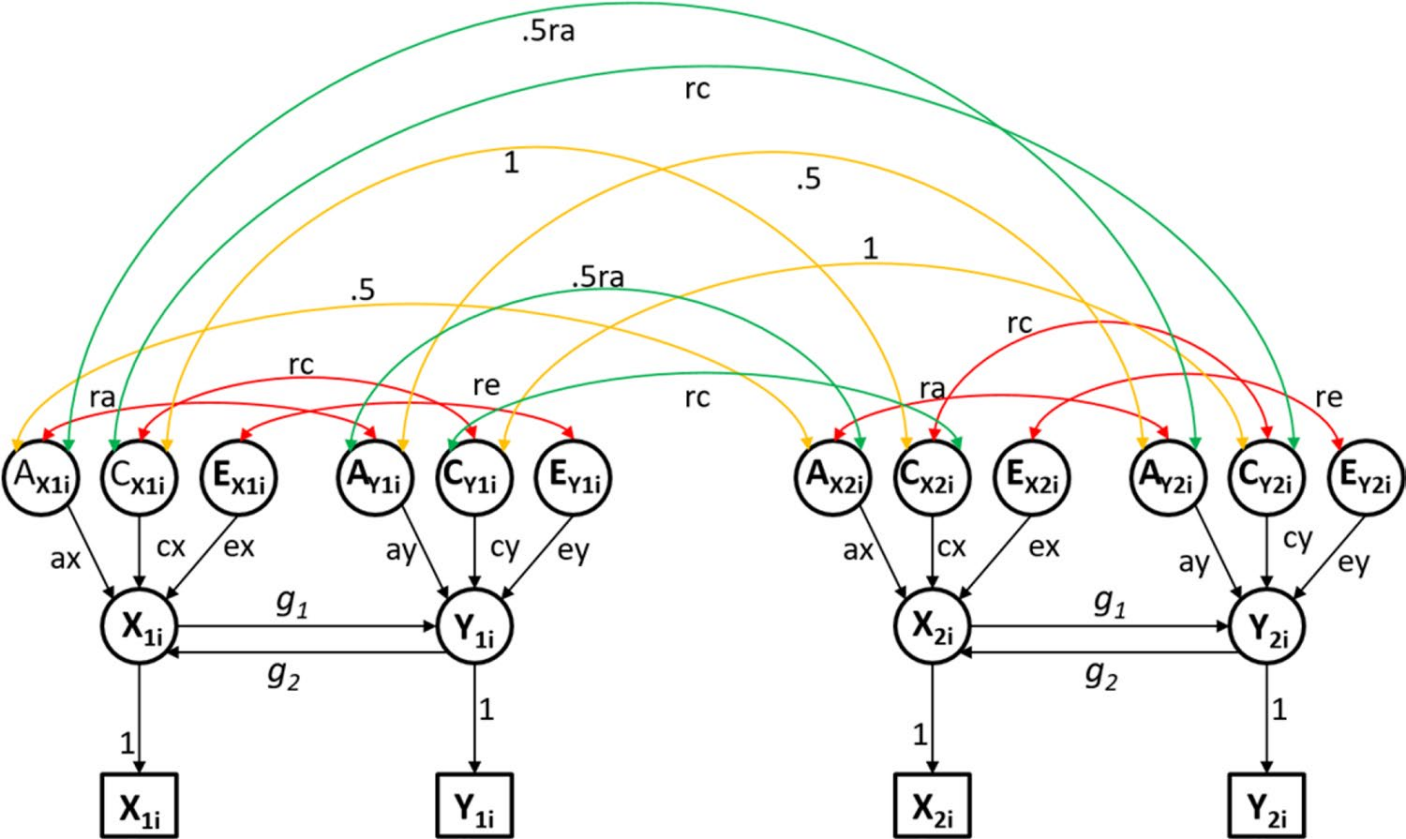
Path diagram representing the Direction of Causation (DoC) twin model given two traits: variable *X* and variable *Y* measured in dizygotic (DZ) twins (twin_1_ and twin_2_). Squares represent observed variables, while circles represent latent variables. *A, C* and *E* stand for additive genetic effects, shared and unique environmental effects, respectively. The double headed arrows represent within/between twins covariances of additive genetic effects (ra), shared environmental effects (rc) and unique environmental effects (re). The cross-twin covariance between additive genetic effects is fixed to .5 (1) for DZ (MZ) twins. DZ (MZ) twins are expected to share on average 50% (100%) of the genetic effects underlying both traits, hence the cross-twin cross-trait covariance is fixed to .5(1) ra for DZ (MZ) twins. Single headed arrows represent causal effects. Note, the model as depicted, is not identified. Typically ra, rc, and re are assumed to be zero in the application of the DoC twin model

### The direction of causation twin model

The Direction of Causation (DoC) twin model (Heath et al. 1993; Gillespie et al. 2003; Verhulst et al. 2012; Verhulst and Estabrook 2012) uses cross-sectional data observed in monozygotic (MZ) and dizygotic (DZ) twins to test causal hypotheses regarding the association between two traits. In contrast to MR, DoC does not necessarily involve a prior hypothesis concerning the causal direction. The path diagram of such a model is shown in Fig. 1, given an exposure variable X and an outcome variable Y, observed in DZ twins.

In Fig. 1X and Y are mutually causally related (parameters g_1_ and g_2_). The traits are subjected to the influence of latent additive genetic (A_X_ and A_Y_), shared (C_X_ and C_Y_) and unique (E_X_ and E_Y_) environmental effects, influences which can be direct or indirect, i.e., via the causal paths (g_1_ and g_2_). As an instance of CTD, this model has the usual assumptions concerning random mating and the absence of genotype-environment interplay (G × E interaction, G × E covariance). The cross-twin correlation of the shared environmental variables is assumed to equal 1 within-trait, and *rc* across traits, regardless of zygosity. By definition, the cross-twin correlation of unique environmental effects is fixed to zero both within and across traits.

The model as depicted in Fig. 1 is not identified; it requires additional restrictions to identify the parameters. By imposing restrictions on the parameters, one can model several alternative hypotheses concerning the observational association between X and Y. The ‘tertium quid’ hypothesis, that a third variable causes both traits, can be tested by constraining the parameters g_1_ and g_2_ to equal zero (i.e., the saturated bivariate model). Uni- and bidirectional causal hypotheses can be tested by fixing to zero the within- and cross-twin cross-trait genetic and environmental correlations (ra, rc, re), and estimating the causal parameters g_1_ and/or g_2_ (i.e., the uni- and the bidirectional causality models). These competing and nested alternative hypotheses can be tested by likelihood ratio (Duffy and Martin 1994; Heath et al. 1993), provided: (1) the two traits differ in their sources of variance (Heath et al. 1993); and (2) there are at least three sources of variance influencing the traits (i.e., A, E, and either C or D, where D denotes dominance) (Mather and Jinks 2012). Given the assumptions mentioned above, we have (Heath et al. 1993):

1 $${{\text{X}}_{{\text{ij}}}}={\text{ }}{{\text{a}}_{\text{X}}}{{\text{A}}_{{\text{Xij}}}}+{\text{ }}{{\text{c}}_{\text{X}}}{{\text{C}}_{{\text{Xij}}}}+{\text{ }}{{\text{e}}_{\text{X}}}{{\text{E}}_{{\text{Xij}}}}+{\text{ }}{{\text{g}}_{\text{2}}}{{\text{Y}}_{{\text{ij}}}}$$

2 $${{\text{Y}}_{{\text{ij}}}}={\text{ }}{{\text{a}}_{\text{Y}}}{{\text{A}}_{{\text{Yij}}}}+{\text{ }}{{\text{c}}_{\text{Y}}}{{\text{C}}_{{\text{Yij}}}}+{\text{ }}{{\text{e}}_{\text{Y}}}{{\text{E}}_{{\text{Yij}}}}+{\text{ }}{{\text{g}}_{\text{1}}}{{\text{X}}_{{\text{ij}}}}$$

where subscript i stands for twin pair, and j for twin (j = 1, 2); X is the exposure variable, and Y is the outcome variable; a, c and e represent the path coefficients relating the phenotypes (X and Y) to the A, C, and E, respectively. The parameters g_1_ and g_2_ are the causal paths accommodating the direct causal effects of X on Y (g_2_) and Y on X (g_2_).

### Standard Mendelian randomization

**Fig. 2:**
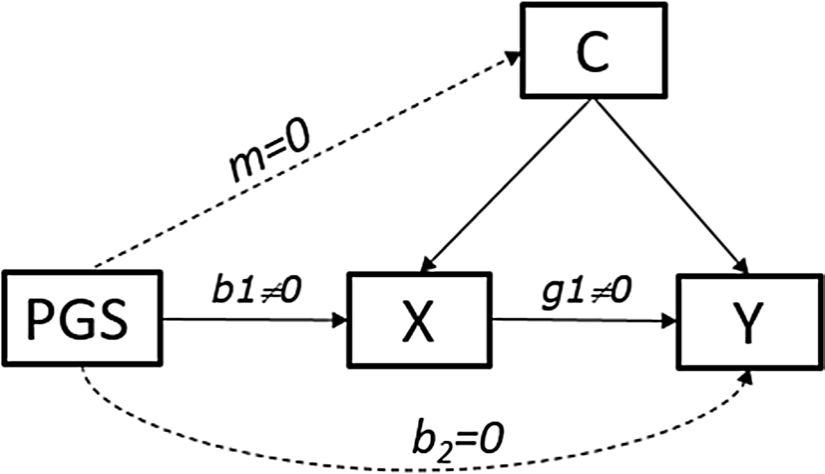
Assumptions in Mendelian randomization. *Hypothesis:* X causes Y. By assumption, the regression coefficients (m and b_2_) associated with the dashed lines are zero. *PGS*-polygenic score, *X*-exposure variable, *Y*-outcome variable, *C-*confounders

The MR model is an instrumental variable regression model, which employs genetic variants as instrumental variables to test causal hypotheses regarding the association between an exposure and an outcome (Smith and Hemani 2014; Burgess and Thompson 2015). Here we assume that the instrument is a polygenic score (PGS). Three assumptions must hold for a genetic variant to be a valid instrumental variable, as shown in Fig. 2. *Assumption 1:* The genetic instrument (PGS) is robustly associated with the exposure variable X (b_1_ ≠ 0 in Fig. 2); *Assumption 2:* PGS is independent of confounders C (m = 0; PGS ⊥ C); *Assumption 3:* PGS is independent of the outcome variable Y conditional on the exposure X and confounders C (b_2_ = 0; PGS ⊥ Y | X, C).

**Fig. 3:**
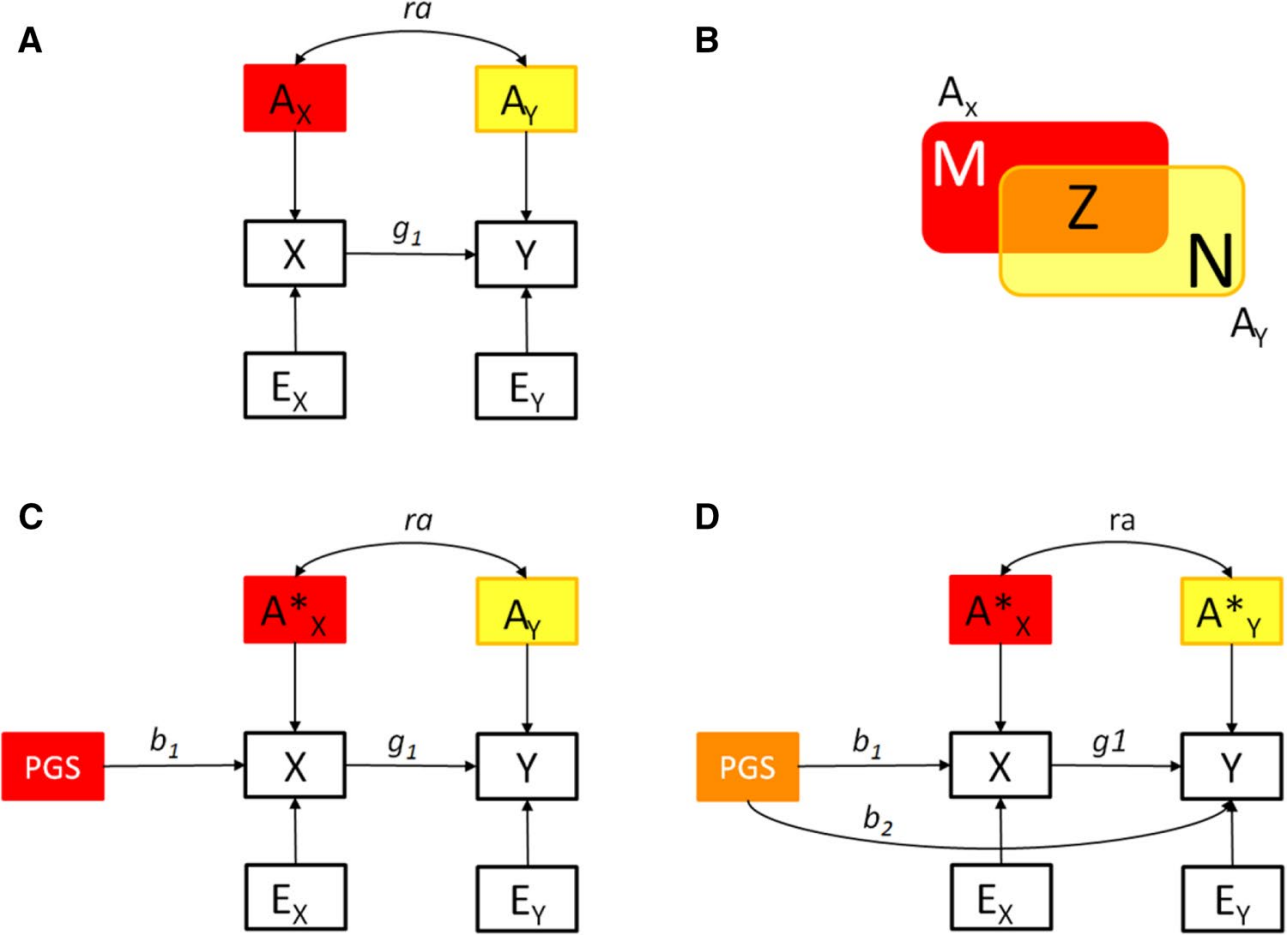
A closer look at the ‘no pleiotropy’ assumption. **A** X directly influences Y (parameter g_1_). In addition, the additive genetic variables A_X_ and A_Y_ are correlated (parameter ra). **B** The set of loci M underlies the variance of A_X_, but does not contribute directly to the variance of A_Y_, i.e., in set theory notation, M = A_X_\A_Y_ [yet note that if X causally influences Y separate powered GWASs of the two traits will associate variants from set M with both X and Y (Solovieff et al. 2013)]. Likewise, N = A_Y_\A_X_, i.e., the set of loci N contributing to the variance of A_Y_ but not to the variance of A_X_. Z represents the intersection of A_X_ and A_Y_, that is, the set of loci Z underlies both A_X_ and A_Y_, i.e., Z = A_Y_ ⋂ A_X_. Note that the set Z may contain pleiotropic loci, where the pleiotropy is due to direct effects or due to linkage disequilibrium; **C** The MR model with a polygenic instrument (PGS) and ‘no pleiotropy’. PGS is associated with X (parameter b1), but is assumed to have no direct influence on the outcome Y. This model holds only if the instrument PGS is constructed on the basis of a subset of variants from set M. In the presence of PGS, A*_X_ is a residual (in the regression of X on PGS). **D** MR with pleiotropic genetic instrument. In this model, the PGS is constructed on the basis of a sample of genetic variants taken from set Z. The parameter b_2_ accommodates the fact that the set of variants used to construct PGS underlies the variance of both A_X_ and A_Y_. The no pleiotropy assumption implies b_2_ = 0

In MR, the third assumption pertains to possible pleiotropic effects of the instrument (PGS), or to the likelihood of including variant(s) in linkage disequilibrium with variants affecting the outcome. In practice, the ‘no pleiotropy’ assumption may be violated, particularly when the instrument is a PGS combining the effects of multiple genetic variants (note that a single variant with pleiotropic effects in principle renders the polygenic score invalid as an instrumental variable). This core MR assumption is illustrated in Fig. 3 where we consider several MR models with and without pleiotropic instruments, and pinpoint the definition of the no pleiotropy assumption.

Among the methods of causal effect estimation in the standard MR are the two-stage least squares (2SLS) and the ratio of coefficients methods. In 2SLS, first, the instrumental variable (e.g., the polygenic score) is used to predict the exposure X, and second, the outcome is regressed on the predicted values of X. In the ratio of coefficients method, the causal effect is computed as a ratio of regression coefficients, with the numerator obtained in the regression of the outcome on the instrument, and the denominator obtained in the regression of the exposure on the instrument [see (Burgess and Thompson 2015)]. Both methods are based on least squares estimation, and are expected to yield equivalent results in MR studies involving a single instrumental variable (Burgess and Thompson 2015).

The standard MR model can also be fitted in a single step as a structural equation model as depicted in Fig. 3 (panel C), with maximum likelihood (ML) estimation [see (Kohler et al. 2011)]. The causal parameter ${\widehat{g}}_{1}$ in Fig. 3c can be tested by the means of a likelihood ratio or a Wald test.

### Integrating Mendelian Randomization with the Direction of Causation twin model (the MR-DoC model)

**Fig. 4:**
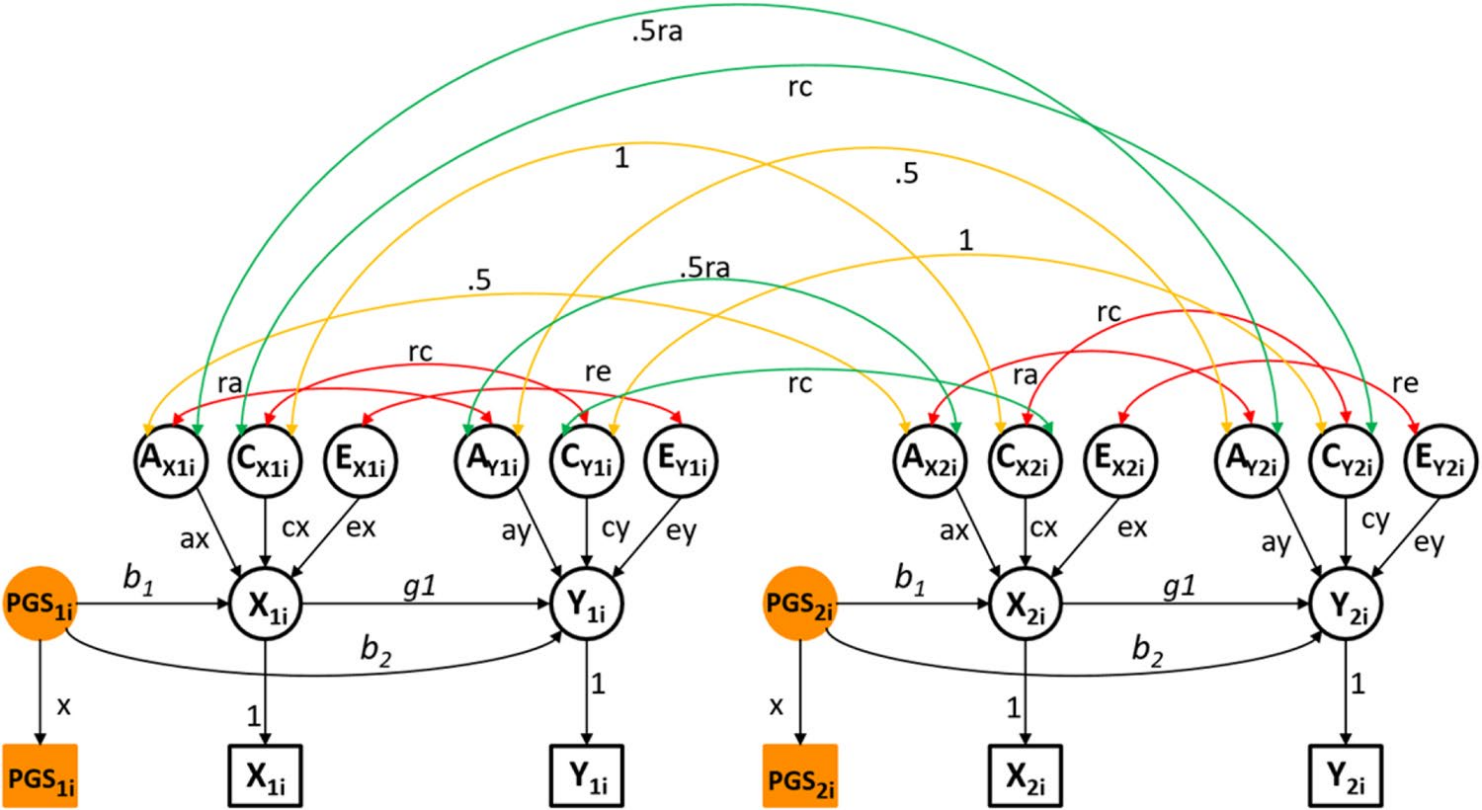
Path diagrammatic representation of the MR-DoC model in DZ twins. The parameter x equals the standard deviation of the observed instrument, i.e., PGS in the circle is standardized. The model as depicted is not identified (see Table 1)

In observational studies, the Mendelian Randomization and the Direction of Causation twin model offer some traction in testing a hypothesized direction of causality. As demonstrated below, the combined MR-DoC model has definite advantages over the individual approaches, in terms of power and assumptions. Figure 4 displays a path diagram of the MR-DoC model. Note that, the model as depicted is not identified. We consider the issue of identification below.

The MR-DoC model is based on the following regression model:

3 $${{\text{X}}_{{\text{ij}}}}={\text{ }}{{\text{a}}_{\text{X}}}{{\text{A}}_{{\text{Xij}}}}+{\text{ }}{{\text{c}}_{\text{X}}}{{\text{C}}_{{\text{Xij}}}}+{\text{ }}{{\text{e}}_{\text{X}}}{{\text{E}}_{{\text{Xij}}}}+{\text{ }}{{\text{b}}_{\text{1}}}{\text{PG}}{{\text{S}}_{{\text{ij}}}}$$

4 $${{\text{Y}}_{{\text{ij}}}}={\text{ }}{{\text{a}}_{\text{Y}}}{{\text{A}}_{{\text{Yij}}}}+{\text{ }}{{\text{c}}_{\text{Y}}}{{\text{C}}_{{\text{Yij}}}}+{\text{ }}{{\text{e}}_{\text{Y}}}{{\text{E}}_{{\text{Yij}}}}+{\text{ }}{{\text{g}}_{\text{1}}}{{\text{X}}_{{\text{ij}}}}+{\text{ }}{{\text{b}}_{\text{2}}}{\text{PG}}{{\text{S}}_{{\text{ij}}}}$$

where i stands for twin pairs, and j for twin (j = 1, 2). The vector of parameters is **θ** = [ra, rc, re, a_x_, c_x_, e_x_, a_y_, c_y_, e_y_, g_1_, b_1_, b_2_, x], where x is the standard deviation of the PGS. Here, and in Fig. 3, the parameter of interest is g_1_, as it concerns the causal effect of exposure X on outcome Y. Note that the MR-DoC model includes the parameter b_2_, representing the pleiotropic effects of PGS on Y (which are usually assumed to be absent, i.e., the central assumption of standard MR of ‘no horizontal pleiotropy’). That is, the parameter b_2_ represents the direct effect of the PGS on the outcome, and accounts for pleiotropy. The estimate of the causal effect g_1_ is unbiased provided the parameter b_2_ is included in the model. Where we refer to a pleiotropic instrument below, we mean that the parameter b_2_ is not zero (parameter b_2_ ≠ 0). Using ML estimation, we can test hypotheses concerning the parameters by means of a likelihood ratio or Wald test.

### Model identification

Model identification concerns the question whether the observed data provide sufficient information to yield unique estimates of the unknown parameters collected in the vector **θ** (Bollen and Bauldry 2010). In the present case, the observed information is summarized in the MZ and DZ (6 × 6) covariance matrices. We assume that the phenotypic means are equal in the MZ and DZ and are equal in the twin 1 and twin 2 members (this is obviously testable). As the parameterization of the means has no bearing on the identification of the covariance structure model, we do not consider them in addressing identification.

Local identification is evaluated at a given set of parameters, say **θ**_**a**,_ and implies there are no points in the vicinity of point **θ**_**a**_ in the parameter space leading to the same expected covariance matrices Σ_MZ_(**θ**_**a**_) and Σ_DZ_(**θ**_**a**_) (Bollen and Bauldry 2010; Bekker et al. 2014). We evaluated local identification using symbolic algebra in Maple (Morgan et al. 2005). Derks et al. (Derks et al. 2006) previously used this method in the context of twin modeling. Using Maple, we checked whether the rank of the Jacobian matrix is full column rank. The Jacobian matrix contains the first order derivatives of the elements in the matrices Σ_MZ_(**θ**_**a**_) and Σ_DZ_(**θ**_**a**_) with respect to the free parameters. If the Jacobian is not full column rank, we require additional parameter constraints (on the elements in the parameter vector **θ**_**a**_). Having established local identification in this manner, we proceeded to address the question of resolution by considering the statistical power to estimate the parameters of interest.

We considered identification in seven models given in Table 1. Model 1, in which all parameters are estimated freely, is not identified. However, constraining to zero any of the parameters re (Model 2), b_2_ (Model 3) or rc (Model 4) renders the model identified. All parameters are identified if the two traits differ with respect to their ACE model. This is the case when the exposure is an ACE trait and the outcome (conditional on the exposure) is an AE trait (implying the parameters c_y_ and rc equal zero, Model 6). Note that in this case Y (unconditional) is characterized by shared environmental effects on Y (transmitted from C_X_, through the g_1_ path) (De Moor et al. 2008). That is, part of variation in Y is attributable to shared environmental factors that contribute to X. These shared environmental effects specific to the exposure identify the parameter g_1_ as they can be related with Y only via the causal path. Furthermore, we found that MR-DoC is not identified when the traits’ variances are limited to two sources (e.g., X and Y are both AE traits, Model 5), or when the exposure is an AE trait and the outcome is an ACE trait (Model 7). Fixing the parameter re to 0 is a constraint commonly employed in the MZ twin intra-pair differences model (Kohler et al. 2011) and in the discordant twin design. In other words, it is assumed that there is no latent confounding from unique environmental sources. That is, the unique environmental component influencing the exposure, influences the outcome only via its effect on the exposure, but not directly. The following results are based on the model with this identifying constraint in place, i.e., re = 0.

**Table 1:** Parameter constraints that render identified the model in Fig. 4

Model	x	a_X_	c_X_	e_X_	a_Y_	c_Y_	e_Y_	ra	rc	re	b_1_	b_2_	g_1_	Identified?
1	fr	fr	fr	fr	fr	fr	fr	fr	fr	fr	fr	fr	fr	No
2	fr	fr	fr	fr	fr	fr	fr	fr	fr	0	fr	fr	fr	Yes
3	fr	fr	fr	fr	fr	fr	fr	fr	fr	fr	fr	0	fr	Yes
4	fr	fr	fr	fr	fr	fr	fr	fr	0	fr	fr	fr	fr	Yes
5	fr	fr	0	fr	fr	0	fr	fr	0	fr	fr	fr	fr	No
6	fr	fr	fr	fr	fr	0	fr	fr	0	fr	fr	fr	fr	Yes
7	fr	fr	0	fr	fr	fr	fr	fr	0	fr	fr	fr	fr	No

Note ‘fr’ indicates that the parameter is estimated, ‘0’—that the parameter is constrained to equal 0

### Power calculations and the type I error rates

**Fig. 5:**
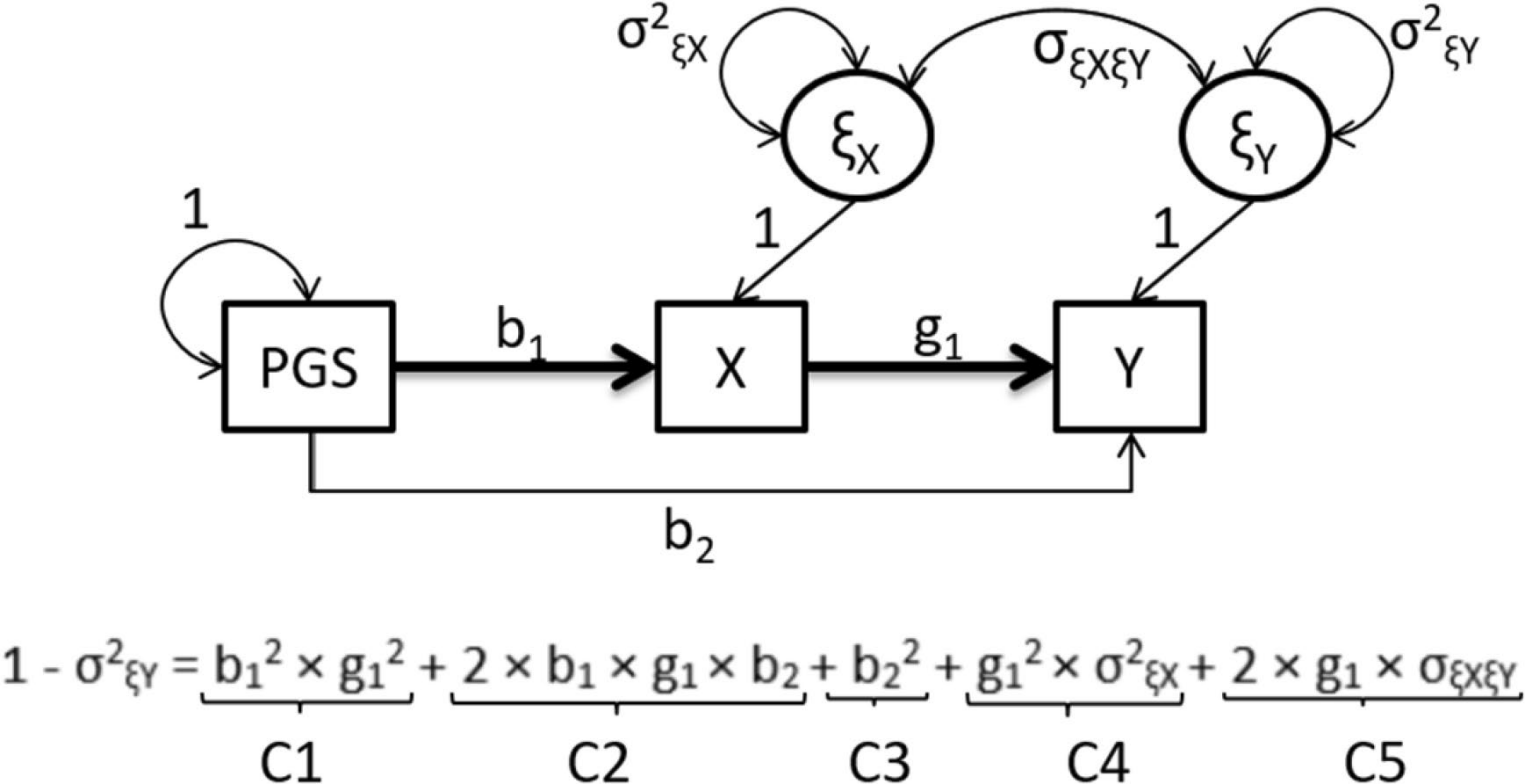
Effect size calculation for the power analyses. *PGS-*polygenic score, *X*-exposure variable, *Y*-outcome variable, *ξ*_*X*_-residual X, *σ*^*2*^_*ξX*_-variance in the exposure, not explained by the instrumental variable PGS, *ξ*_*Y*_-residual Y, *σ*^*2*^*ξ*_*Y*_-variance in Y not explained by the MR model, *σ*_*ξXξY*_-the covariance between ξ_X_ and ξ_Y_, *b*_*1*_-regression coefficient in the MR regression of the exposure X on the instrument, *b*_*2*_-regression coefficient in the MR regression of the outcome on the PGS, *g*_*1*_-the causal effect of X on Y. Note that all parameters are estimated simultaneously in this MR model, hence e.g., the b_2_ parameter estimated in the MR regression will not equal the regression coefficient obtained in the OLS regression of Y on PGS (the latter regression coefficient captures both direct and indirect effect of the PGS on Y)

We used exact data simulation to create data that fit a given identified model exactly [i.e., the observed covariance matrices equalled the expected covariance matrices exactly; see (Van Der Sluis et al. 2008)]. That is, we simulated normal data on the exposure, outcome and polygenic scores in 2000 MZ and DZ twin pairs, given the scenarios described in the Supplementary Tables S1 and S6. The polygenic score was generated as a continuous, normal variable. We considered PGS with effect sizes of 5 and 10% explained variance of the exposure. We fitted the true model, thus retrieving the parameter values exactly as specified, and then dropped the parameters of interest to assess the power in the standard way using the non-central χ^2^ distribution, with noncentrality parameter (NCP) λ. We adopted an alpha of .05 throughout. Data were simulated in R using the MASS library function mvrnorm() with the empirical = TRUE option (R-Core-Team 2015). The MR-DoC model was fitted to the population covariance matrices in OpenMx (Neale et al. 2016). We used the R-package AER (Kleiber and Zeileis 2008) to conduct 2SLS estimation in the standard MR using the sample of twin 1 data, effectively a sample of unrelated individuals. We used the mRnd power calculator to calculate the power of the 2SLS procedure (Brion et al. 2013). We chose effect sizes by considering the decomposition of the variance of the outcome Y, as illustrated in Fig. 5. That is, given that the outcome Y is standardized (σ^2^_Y_ = 1), we considered the components making up the explained variance, i.e., 1−σ^2^ξ_Y_, as a function of the regression parameters b_1_, b_2_, g_1_, the variances of the residuals ξ_X_ and ξ_Y_ (parameters σ^2^_ξX_ and σ^2^_ξY_), and the covariance between the residuals ξ_X_ and ξ_Y_ (parameter σ_ξXξY_).

We varied (a) the strength of the instrumental variable, defined as the percentage of variance explained in the exposure X by instrumental PGS; (b) the variance of ξ_X_ (residual X), i.e., parameter σ^2^_ξX_, representing the percentage of variance in the exposure, not explained by the instrumental variable; (c) the variance in ξ_Y_ (residual Y), i.e., parameter σ^2^ξ_Y_, representing the percent of variance in Y not explained by the MR model; and (d) the covariance between ξ_X_ and ξ_Y_ (parameter σ_ξXξY_). Using the path tracing rules, we can distinguish five components of variance (C1 to C5, Fig. 5) that involve the parameters of interest g_1_ (the causal effect) and b_2_ (representing the direct—pleiotropic—effect of the instrument on the outcome). In all scenarios, the total explained variance of the outcome Y equalled 10%. The parameter values used in simulations are included in the Supplementary Tables S1 and S6. To provide an indication on the potential gains in power conferred by our approach relative to a standard MR analysis of data obtained in unrelated individuals, we report the number of unrelated persons required to attain equivalent power as MR-DoC based on 2000 twin pairs.

We used Monte Carlo Simulations to evaluate type I error rate across all scenarios considered above. We simulated 10,000 samples under the null model of no causal effect of the exposure on the outcome variable (parameter g_1_ equalled 0). Each sample consisted of 2000 twin pairs. The type I error was calculated as the percentage of datasets in which the null hypothesis was incorrectly rejected given two significance thresholds, 0.05 and 0.01.

## Results

### Parameter recovery in standard MR and MR-DoC with valid or invalid (i.e., pleiotropic) instrumental variables (given re = 0)

Table 2 displays results obtained using non-pleiotropic (i.e., Fig. 3, b_2_ = 0), or pleiotropic (i.e., b_2_ ≠ 0, see Fig. 4) instrumental variables in the causal effect estimation. Given b_2_ = 0, results indicate that all estimation methods recover the true parameter value (scenario S1, Table 2). As is to be expected, b_2_ ≠ 0 leads to biased estimates of the causal effect when employing standard MR methods (e.g., 2SLS or ratio of coefficients). MR-DoC recovers correctly the true parameter values (scenario S2, Table 2). Finally, we checked parameter recovery when the instrument has pleiotropic effects, but there is no causal effect of the exposure X on outcome Y (i.e., b_2_ ≠ 0 and g_1_ = 0; scenario S3, Table 2). Results showed that standard MR detects a causal effect (when in truth there is none), while MR-DoC does not. We remind the reader that we have set re = 0 to obtain these results.

**Table 2:** Results of the comparison of the standard MR in analysis of unrelated individuals and MR-DoC twin model

Scenario	Parameter values (simulated)	TSLS/Two-sample MR/MR as SEM (estimated)	MR-DoC (estimated)
1. Non-pleiotropic instrument and non-zero causal relationship between exposure and outcome	b_1_ = 0.3162 b_2_ = 0 g_1_ = 0.1838	b_1_ = 0.3162 g_1_ = 0.1838	b_1_ = 0.3162 b_2_ = 0 g_1_ = 0.1838
2. Pleiotropic instrument and non-zero causal relationship between exposure and outcome	b_1_ = 0.3162 b_2_ = 0.1599 g_1_ = 0.1265	b_1_ = 0.3162 *g*_*1*_ = *0.6324*	b_1_ = 0.3162 b_2_ = 0.1599 g_1_ = 0.1265
3. Pleiotropic instrument and no causal relationship between exposure and outcome	b_1_ = 0.3162 b_2_ = 0.3162 g_1_ = 0	b_1_ = 0.3162 *g*_*1*_ = *1.00*	b_1_ = 0.3162 b_2_ = 0.3162 g_1_ = − 4.86e-08

Additive genetic (A), shared environmental (C) and unique environmental (E) effects contributed to the variance of both the exposure (a^2^_X_ = 0.5, c^2^_X_ = 0.2, e^2^_X_ = 0.3) and the outcome (a^2^_X_ = 0.2, c^2^_X_ = 0.1, e^2^_X_ = 0.7) variable, and genetic and shared environmental factors contributed to the correlations between the traits (r_ξXξY_ = 0.2). Incorrectly estimated parameter values are in italics

### Power and type I error rates

**Fig. 6:**
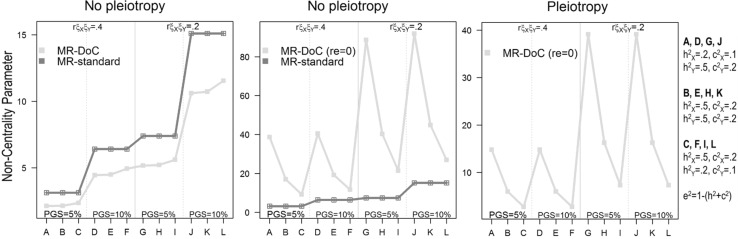
Results given a *non-pleiotropic* instrument (parameter b_2_ = 0; left and middle panels), and given a *pleiotropic* instrument (parameter b_2_ ≠ 0; right panel). Fitting the model with the parameter g_1_ freely estimated, and with the parameter g_1_ constrained to equal zero provided the Non-Centrality Parameter (NCP). The standard MR is based on two-stage least squares in a sample of 4000 unrelateds. The MR-DoC twin model used maximum likelihood and a sample of 2000 twin pairs. *r*_*ξXξY*_ residual exposure-outcome correlation, *PGS* polygenic score

Using Monte Carlo simulations we established that the type I error rate is correct (see Supplementary Tables S2, S4 and S7). Figure 6 (and Supplementary Tables S3, S5 and S8) displays the results pertaining to the power to detect the causal effect in standard MR and in MR-DoC.

With a valid instrumental variable (no pleiotropy) and all parameters freely estimated (including the parameter re, Table S3), the main factors that influence MR-DoC’s power are: (a) instrument’s strength; (b) the genetic covariance structure of X (exposure) and Y (outcome); and (c) the magnitude of the residual X–Y correlation. As expected, increasing instrument’s strength increases power. For instance, with an ACE trait as the exposure variable (a^2^_X_ = .5, c^2^_X_ = .2, e^2^_X_ = .3), an outcome variable having roughly the same mode of inheritance (a^2^_Y_ = .5, c^2^_Y_ = .2, e^2^_Y_ = .3), and a residual correlation of r_ξXξY_ = .2, the power of the MR-DoC equals .627 and .905 when the instrument explains 5 and 10% of the variance in the exposure, respectively. However, contrary to the standard DoC literature, having traits with similar genetic covariance structure has no bearing on MR-DoC’s power to detect the causal effect. This is the case for instance, if both the outcome and exposure are ACE traits. Power is the highest when the outcome variable has a lower heritability than the exposure variable. For example, power increases from .622 in Scenario S1G (with a 50% heritable outcome and a 20% heritable exposure) to .658 in Scenario S1I (with a 20% heritable outcome variable and a 50% heritable exposure; Table S3). Finally, increasing X–Y residual correlation reduces MR-DoC’s power. For instance, with an outcome and an exposure having roughly the same mode of inheritance, and an instrument explaining 5% of the variance in the exposure, MR-DoC’s power drops from .627 (Scenario S1H, Table S3) to .312 (Scenario S1B) when the residual correlation increases from r_ξXξY_ = 0.2 to r_ξXξY_ = 0.4.

Given equal N, standard MR (4000 unrelateds) has larger power than MR-DoC (4000 twins) only when b_2_ = 0 and re ≠ 0 (Fig. 6, left). Yet, assuming data collected in twins are readily available, greater statistical power is available in the MR-DoC model (than from reducing the twin pairs to singletons and resorting to standard MR, Table S5).

We also calculated the required N of unrelated individuals to achieve the same power as 2000 twin pairs (Table 3). We found the yield of MR-DoC substantial if the parameter re was fixed to zero (as simulated, Fig. 6, middle). For example, with a sample of 2000 twin pairs, MR-DoC yields a NCP λ of 38.68 given an instrument explaining 10% of the variance in the exposure, a residual correlation of r_ξXξY_ = 0.2, an exposure variable with low heritability (a^2^_X_ = 0.2, c^2^_X_ = 0.1, e^2^_X_ = 0.7), and a moderately heritable outcome (a^2^_Y_ = 0.5, c^2^_Y_ = 0.2, e^2^_Y_ = 0.3). Standard MR needs about 56 737 unrelated individuals to achieve equivalent power (Table 3).

**Table 3:** The number of unrelated individuals (N) required by MR-standard to achieve the same NCP as MR-DoC in 2000 twin pairs

Scenario (S1)	N required by MR standard to achieve the same NCP as MR-DoC with **re** = **0**
A	56,737
B	24,945
C	13,472
D	28,515
E	13,480
F	8206
G	53,774
H	24,440
I	13,013
J	13,558
K	13,239
L	7954

The number of unrelateds was estimated based on the NCP obtained by fitting the standard MR as a structural equation model, with estimation of the causal effect based on maximum likelihood (similar to MR-DoC)

Given b_2_ ≠ 0 and re = 0, MR-DoC’s power increases with: (a) decreasing X-Y residual correlation, and (b) decreasing residual variance of the exposure (Fig. 6, right; see for details, Tables S2 and S5). Regarding the former, power is always larger when the association between the exposure and the outcome is largely causal in nature (i.e., when the residual correlation drops from r_ξXξY_ = 0.4 to r_ξXξY_ = 0.2). Regarding the latter, power is always greater in scenarios where the exposure has low heritability. For instance, the NCP λ increases from 7.29 to 39.1 when the heritability of the exposure decreases from 50% (S2I) to 20% (S2J, Table S8). The same pattern of results was observed when b_2_ = 0 and re = 0 (see Fig. 6, middle, and Table S5). Lowering the parameter a_x_ (reducing the background additive genetic variance of the exposure) will reduce the residual variance in the regression of the exposure on the outcome and this increases the instrument strength. This will render the role of g_1_ (the causal effect) in connecting X and Y relatively greater. This in turn increases the power to detect g_1_. We note that the same applies when lowering the parameter c_x_. Note that the instrument’s strength has no longer a bearing on power when b2 ≠ 0, i.e., when it affects the outcome both directly (parameter b_2_) and indirectly, via the exposure (parameter b_1_). For instance, 2000 twin pairs yield an NCP λ of 5.98 given two traits having identical variance components (a^2^_X_ = a^2^_Y_ = 0.5; c^2^_X_ = c^2^_Y_ = 0.2; e^2^_X_ = e^2^_Y_ = 0.3), and a large residual correlation (r_ξXξY_ = 0.4), regardless of whether the instrument explains 5 or 10% of the variance in the exposure (Scenarios S2B and S2E, Table S8). As mentioned above, contrary to the standard DoC literature [see e.g., (Heath et al. 1993)], MR-DoC does not require the exposure and the outcome variable to have radically different covariance structures to ensure sufficient power to test unidirectional causal hypotheses.

## Discussion

Our aim was to integrate MR and the classical twin model to render testable the MR’s strong assumption that the instrumental variable has no direct effect on the outcome, conditional on the exposure and confounders. We showed that, with standard MR methods, violations of this assumption readily lead to biased causal effect estimates, or may even yield spurious false positives. MR-DoC correctly detects causal effects and provides accurate parameter estimates even if the instrument is pleiotropic. With traits that have the same covariance structure (e.g., when both the outcome and exposure are ACE traits), the weaker assumption (used also in the MZ discordant twin design) that there is no latent confounding from unique environmental sources of variation may be needed (see Table 1). However, the effect of re = 0 can be studied by fixing re to various values with the objective of gauging the sensitivity of the results to the value of re.

Note that the assumption re = 0 is not required with traits that have different covariance structures (i.e., with an ACE trait as the exposure and an AE trait as an outcome one may estimate re, b_2_ as well as g_1_, see Fig. 4). We did not consider extensively this scenario because although formally identified, this model has poor resolution (but see Supplementary Table S9 and Figure S1 for results of a small power study given parameter r_c_ = 0). That is, employing the model would require pooling the genotype data from multiple twin registries in a mega-analysis to ensure adequate power.

Aside from providing the means to relax the ‘no pleiotropy’ assumption, the MR-DoC twin model confers several other advantages in understanding causal relationships between exposures and outcomes. First, MR-DoC provides full statistical description of the observed exposure-outcome relationship, allowing one to disentangle the causal effect of the exposure on the outcome, from potential pleiotropic effects of the instrumental variable, as well as from the contribution of other genetic and environmental factors to the variances and the covariance of the two traits. Second, the twin data provide sufficient information to estimate the direct path from the instrument to the outcome (i.e., the parameter b_2_ in Fig. 4). Importantly, this path captures not only pleiotropic effects, but also possible effects of other variants affecting the outcome which are in linkage disequilibrium with the instrument. Third, our approach opens up the possibility of using strong genetic instrumental variables in the form of polygenic scores. This is generally desirable in the standard MR design, as the strength of the valid instrumental variable has a bearing on the precision of the causal estimate [i.e., with weak instruments the estimate tends to approach the observed OLS exposure-outcome association (Bound et al. 1995)], and on the distribution of the causal effect estimate [the weaker the instrument, the more skewed the distribution; see Fig. 7.1 in (Burgess and Thompson 2015)]. As a consequence, significance tests and the construction of the confidence intervals, which rely on asymptotic normality, are no longer accurate. Consequently, tests may suffer inflated type I error rates (Burgess et al. 2015). In addition, strong instruments are desirable from the perspective of power, as our results showed [consistent with the literature, see, e.g., (Burgess et al. 2015)].

Interestingly, the strength of the instrument (i.e., defined in terms of percentage of explained variance in the exposure), has no bearing on the power when the instrument has pleiotropic effects, given that these effects are correctly modeled (in MR-DoC). The reason for this is that the exposure no longer features as a full mediator variable in the presence of a direct path between the instrument and the outcome. Correspondingly, the misfit due to dropping the causal parameter from the analysis is attenuated by the presence of the parameter b_2_ in the model. That is, fixing the parameter g_1_ to zero will largely bias the parameter b_2_, but will not affect the between twin covariance matrix (as is the case in the standard MR design where the sole path from the instrument to the outcome is via the exposure). Stated otherwise, the bias in b_2_ will be greatest, leaving ra (Ax, Ay) and rc (Cx, Cy) relatively unaffected, regardless of how strong the instrument is. In this circumstance, the power to detect the causal effect will primarily depend on the magnitude of the residual correlation between the two traits and, to a lesser extent, on their modes of inheritance.

An important caveat to consider when employing the MR-DoC model concerns the issue of measurement error. As extensively discussed in the DoC literature (Heath et al. 1993; Kohler et al. 2011) the estimates of the model parameters including the estimate of the parameter of interest g_1_ will be biased when there is measurement error in the exposure X. This problem can be circumvented by employing multiple indicators of the exposure [i.e., see for details (Heath et al. 1993; Kohler et al. 2011)], or by including prior information on the reliability of the exposure.

MR-DoC is tailored for the readily available datasets collected worldwide on more than 1.5 million twins at the Twin and Family Registries [see (Hur and Craig 2013) for details on these rich resources of genotypic and phenotypic data]. We showed that twin data correctly detect causation and estimate effect sizes even in the presence of pleiotropy. Although primarily developed to address the ‘no pleiotropy’ assumption, our results demonstrate that MR-DoC has greater statistical power than standard MR analysis in unrelated individuals if the parameter re is zero (as fixed to zero in the model). With re fixed to zero, dropping the causal parameter g_1_ from the model greatly impacts all paths connecting the exposure and the outcome, both within and across twins (parameters ra and rc), creating a large discrepancy between the observed and the expected covariance matrix. This misfit is evident throughout the covariance matrix. Consequently, in some specific circumstances, testing causal hypotheses requires tens of thousands of unrelated individuals for one to achieve the same power as that conferred by several hundreds of twin pairs. It should be noted that unlike MR methods (Bowden et al. 2015, 2016) or asymmetry tests (Pickrell et al. 2016) based on summary statistics (methods which require GWAS results for both the exposure and the outcome variables), the approach proposed here only requires GWAS results for the exposure variable (i.e., MR-DoC needs a genetic instrument for X - constructed based on a GWAS of the exposure, and phenotypic measures for X and Y). With MR-DoC and these rich phenotype resources - ranging from personality, diet and lifestyle to disease and psychiatric traits [see e.g.,(Hur and Craig 2013)]—collected at Twin and Family registries, the availability of genetic instruments robustly associated with the exposure remains the main limiting factor in addressing causal questions in non-experimental settings.

As presented here, MR-DoC is limited to twin data. Yet twin registries often have available information on additional family members (Hur and Craig 2013; Kaprio 2013; Skytthe et al. 2012; van Beijsterveldt et al. 2013; Willemsen et al. 2013). It is important to note that the method developed here can be generalized to sib data and within family tests. Additionally, as not all cohort studies necessarily include related individuals, we aim to expand the model, to accommodate distantly related individuals by using Genetic Relationship Matrices (GRMs) based on average allelic correlations (where the alleles are defined at the measured single nucleotide polymorphisms). We anticipate that these extensions will further increase statistical power and robustness to assumption violation (Keller et al. 2010; Posthuma and Boomsma 2000). Second, throughout the paper we assumed that the mating is random, there is no genotype-environment correlation, and no genotype by environment interaction. Indeed, assumption violation may also arise because the mating is assortative (Burgess and Thompson 2015), or because there are other plausible paths from the instrument to the outcome (except direct paths, or indirect, via the exposure), for example, via confounders affecting both traits, i.e., implying genotype by environment interaction or genotype by environment correlation. However, we note these effects may be captured by the MR-DoC twin model with appropriate experimental designs (Keller et al. 2010; Plomin et al. 2013; Posthuma et al. 2003). On a cautionary note, although valid strong instruments are desirable in MR from the perspective of power, making up the polygenic score based on variants of unknown function should limit the testable hypotheses to whether the model is consistent or not with a direct causal effect from the exposure to the outcome [as pointed out by Burgess et al. (2014)]. While we considered the use of MR-DoC with polygenic scores, our conclusions also hold in scenarios where a genetic variant with known function is used as the instrument, which would improve the biological interpretation of the causal effect.

In conclusion, by integrating Mendelian Randomization with the Direction of Causation twin model, we developed a model that allows one to test and relax the strong ‘no pleiotropy’ assumption employed by standard MR. This approach therefore allows one to employ strong instrumental variables in the form of polygenic scores, guarding against weak instrument bias, and increasing the power to detect causal effects of exposures on potential outcomes.

We believe causal modeling in random samples (i.e., without experimental control) to be a considerable challenge, and subject to great interest and many developments [see e.g., (Burgess et al. 2017)]. It is unlikely—in the foreseeable future—that any single method or approach will be definite; it is more likely that the robust demonstration of causality will require evidence from multiple models and approaches (each with its own weaknesses and strengths). We believe that the approach presented here will make a valuable contribution to this undertaking. We anticipate that MR-DoC will enhance and extend MR’s range of applications, and increase the value of the large cohorts collected at twin registries as they correctly detect causation and estimate effect sizes even in the presence of pleiotropy.

## Electronic supplementary material

Below is the link to the electronic supplementary material.

Supplementary material 1 (DOCX 446 KB)

## Footnotes

1 Bidirectional MR, as presented in the literature, involves two unidirectional sequential tests: Model 1 representing the hypothesis1 X causes Y (parameter g_1_ estimated), and Model 2 representing the hypothesis2 Y causes X (parameter g_2_ estimated, see Fig. 1 below). A true bidirectionality test involves estimating simultaneously the g_1_ and g_2_ parameters.
