# Supplementary material for: Extending Causality Tests with Genetic Instruments: An Integration of Mendelian Randomization with the Classical Twin Design
Source: Behav Genet. 2018 Jun 7;48(4):337–49. doi: 10.1007/s10519-018-9904-4 (PMC6028857; doi:10.1007/s10519-018-9904-4)

**Supplementary Tables and Figures**

Table S1: Scenarios and parameter values used to generate data for assessing the power of the MR–DoC twin model, given a *non-pleiotropic* instrumental variable (parameter b_2_=0). We varied the strength of the instrument, (i.e., the Polygenic Score explained either 0.05 or 0.1 proportion of the variance in the exposure), the proportion of variance explained in the exposure (X) and the outcome (Y) variable by additive genetic (h^2^_X_=σ^2^_Ax_/σ^2^_phX_; h^2^_Y_= σ^2^_Ay_/σ^2^_phY_), shared environmental (c^2^_X_=σ^2^_Cx_/σ^2^_phX_; c^2^_Y_ = σ^2^_Cy_/σ^2^_phY_) and unique environmental (e^2^_X_=σ^2^_Ex_/σ^2^_phX_; e^2^_Y_=σ^2^_Ey_/σ^2^_phY_) factors, and the contribution of the instrumental variable (PGS; parameter b_1_), the causal effect (g_1_) and of the residual correlation between the outcome and the exposure (r_ξXξY_) to the 10% explained variance in the outcome. The effect size (see the five components of variance C1 to C5 in Fig.5) is defined as the percentage of explained variance in the outcome given the chosen parameter values b_1_, b_2_, g_1_, σ_ξXξY_ (covariance of ξX and ξY), σ^2^_ξX_ (residual variance in X), and σ^2^_ξY_ (residual variance in Y).

| Scenario (S1) | Parameter values | Effect size: percentage (%) of variance explained in the outcome (variance component in Fig.5) |
| --- | --- | --- |
| A | PGS = 0.05  h^2^_X_ = 0.2, c^2^_X_ = 0.1, e^2^_X_ = 0.7  h^2^_Y_ = 0.5, c^2^_Y_ = 0.2, e^2^_Y_ = 0.3  r_ξXξY_ = 0.4 | b_1_^2^ × g_1_^2^ = 0.068 (C1)  g_1_^2^ × σ^2^_ξX_ = 1.295 (C4)  2 × g_1_ × σ_ξXξY_ =8.636 (C5) |
| B | PGS = 0.05  h^2^_X_ = 0.5, c^2^_X_ = 0.2, e^2^_X_ = 0.3  h^2^_Y_ = 0.5, c^2^_Y_ = 0.2, e^2^_Y_ = 0.3  r_ξXξY_ = 0.4 | b_1_^2^ × g_1_^2^ = 0.068 (C1)  g_1_^2^ × σ^2^_ξX_ = 1.295 (C4)  2 × g_1_ × σ_ξXξY_ =8.636 (C5) |
| C | PGS = 0.05  h^2^_X_ = 0.5, c^2^_X_ = 0.2, e^2^_X_ = 0.3  h^2^_Y_ = 0.2, c^2^_Y_ = 0.1, e_y_^2^= 0.7  r_ξXξY_ = 0.4 | b_1_^2^ × g_1_^2^ = 0.068 (C1)  g_1_^2^ × σ^2^_ξX_ = 1.295 (C4)  2 × g_1_ × σ_ξXξY_ =8.636 (C5) |
| D | PGS = 0.1  h^2^_X_ = 0.2, c^2^_X_ = 0.1, e^2^_X_ = 0.7  h^2^_Y_ = 0.5, c^2^_Y_ = 0.2, e^2^_Y_ = 0.3  r_ξXξY_ = 0.4 | b_1_^2^ × g_1_^2^ = 0.142 (C1)  g_1_^2^ × σ^2^_ξX_ = 1.278 (C4)  2 × g_1_ × σ_ξXξY_ = 8.579 (C5) |
| E | PGS = 0.1  h^2^_X_ = 0.5, c^2^_X_ = 0.2, e^2^_X_ = 0.3  h^2^_Y_ = 0.5, c^2^_Y_ = 0.2, e^2^_Y_ = 0.3  r_ξXξY_ = 0.4 | b_1_^2^ × g_1_^2^ = 0.142 (C1)  g_1_^2^ × σ^2^_ξX_ = 1.278 (C4)  2 × g_1_ × σ_ξXξY_ = 8.579 (C5) |
| F | PGS = 0.1  h^2^_X_ = 0.5, c^2^_X_ = 0.2, e^2^_X_ = 0.3  h^2^_Y_ = 0.2, c^2^_Y_ = 0.1, e^2^_Y_ = 0.7  r_ξXξY_ = 0.4 | b_1_^2^ × g_1_^2^ =0.142 (C1)  g_1_^2^ × σ^2^_ξX_ = 1.278 (C4)  2 × g_1_ × σ_ξXξY_ = 8.579 (C5) |
| G | PGS = 0.05  h^2^_X_ = 0.2, c^2^_X_ = 0.1, e^2^_X_ = 0.7  h^2^_Y_ = 0.5, c^2^_Y_ = 0.2, e^2^_Y_ = 0.3  r_ξXξY_ = 0.2 | b_1_^2^ × g_1_^2^ = 0.164 (C1)  g_1_^2^ × σ^2^_ξX_ = 3.126 (C4)  2 × g_1_ × σ_ξXξY_ = 6.70 (C5) |
| H | PGS = 0.05  h^2^_X_ = 0.5, c^2^_X_ = 0.2, e^2^_X_ = 0.3  h^2^_Y_ = 0.5, c^2^_Y_ = 0.2, e^2^_Y_ = 0.3  r_ξXξY_ = 0.2 | b_1_^2^ × g_1_^2^ = 0.164 (C1)  g_1_^2^ × σ^2^_ξX_ = 3.126 (C4)  2 × g_1_ × σ_ξXξY_ =6.709 (C5) |
| I | PGS = 0.05  h^2^_X_ = 0.5, c^2^_X_ = 0.2, e^2^_X_ = 0.3  h^2^_Y_ = 0.2, c^2^_Y_ = 0.1, e^2^_Y_ = 0.7  r_ξXξY_ = 0.2 | b_1_^2^ × g_1_^2^ = 0.164 (C1)  g_1_^2^ × σ^2^_ξX_ = 3.126 (C4)  2 × g_1_ × σ_ξXξY_ =6.709 (C5) |
| J | PGS = 0.1  h^2^_X_ = 0.2, c^2^_X_ = 0.1, e^2^_X_ = 0.7  h^2^_Y_ = 0.5, c^2^_Y_ = 0.2, e^2^_Y_ = 0.3  r_ξXξY_ = 0.2 | b_1_^2^ × g_1_^2^ =0.338 (C1)  g_1_^2^ × σ^2^_ξX_ =3.042 (C4)  2 × g_1_ × σ_ξXξY_ =6.619 (C5) |
| K | PGS = 0.1  h^2^_X_ = 0.5, c^2^_X_ = 0.2, e^2^_X_ = 0.3  h^2^_Y_ = 0.5, c^2^_Y_ = 0.2, e^2^_Y_ = 0.3  r_ξXξY_ = 0.2 | b_1_^2^ × g_1_^2^ =0.338 (C1)  g_1_^2^ × σ^2^_ξX_ =3.042 (C4)  2 × g_1_ × σ_ξXξY_ =6.619 (C5) |
| L | PGS = 0.1  h^2^_X_ = 0.5, c^2^_X_ = 0.2, e^2^_X_ = 0.3  h^2^_Y_ = 0.2, c^2^_Y_ = 0.1, e^2^_Y_ = 0.7  r_ξXξY_ = 0.2 | b_1_^2^ × g_1_^2^ =0.338 (C1)  g_1_^2^ × σ^2^_ξX_ =3.042 (C4)  2 × g_1_ × σ_ξXξY_ =6.619 (C5) |

Table S2: Type I error rate for the MR-DoC model given the scenarios described in Table S1 and a *non-pleiotropic* instrumental variable (parameter b_2_ = 0). We simulated 10 000 samples under the null model of no causal effect of the exposure on the outcome variable (parameter g_1_ equalled 0). Each sample consisted of 2000 twin pairs. The type I error was calculated as the percentage of datasets in which the null hypothesis was incorrectly rejected given two significance thresholds, 0.05 and 0.01. In fitting the MR-DoC model we estimate all model parameters (with the parameter re, simulated re=0, freely estimated). Abbreviation: CI – Confidence Interval.

| Scenario (S1) | alpha=0.01 [99% CI] | alpha=0.05 [99% CI] |
| --- | --- | --- |
| A | 0.0093 [0.0070, 0.0121] | 0.0504 [0.0450, 0.0563] |
| B | 0.0091 [0.0069, 0.0119] | 0.0507 [0.0452, 0.0567] |
| C | 0.009 [0.0068, 0.0118] | 0.0489 [0.0435, 0.0548] |
| D | 0.0107 [0.0083, 0.0137] | 0.0512 [0.0457, 0.0572] |
| E | 0.0116 [0.0091, 0.0147] | 0.0509 [0.0454, 0.0569] |
| F | 0.009 [0.0068, 0.0118] | 0.0461 [0.0409, 0.0518] |
| G | 0.0099 [0.0076, 0.0128] | 0.0493 [0.0439, 0.0552] |
| H | 0.0103 [0.0079, 0.0133] | 0.051 [0.0455, 0.0570] |
| I | 0.0081 [0.0060, 0.0108] | 0.048 [0.0427, 0.0538] |
| J | 0.0107 [0.0083, 0.0137] | 0.0511 [0.0456, 0.0571] |
| K | 0.0107 [0.0083,0.0137] | 0.05 [0.0446, 0.0559] |
| L | 0.0099 [0.0076, 0.0128] | 0.05 [0.0446, 0.0559] |

Table S3: Simulation results given a *non-pleiotropic* instrumental variable (parameter b_2_ = 0). We report the power to detect the causal effect g_1_ (given alpha=0.05), and the NCP obtained based on the standard MR with the causal effect estimated using two-stage least squares (N=2000 unrelateds), and based on the MR-DoC twin model (N=2000 twin pairs). In fitting the MR-DoC model we estimate all model parameters (with the parameter re, simulated re=0, freely estimated).

| Scenario (S1) | MR-DoC’s power (NCP)  in 2000 twin pairs | Standard MR’s power (NCP)  in 2000 unrelated individuals  (twin1) | Standard MR’s power (NCP)  in 4000 unrelated individuals |
| --- | --- | --- | --- |
| A | .309 (2.13) | .25 (1.61) | .42 (3.13) |
| B | .312 (2.15) | .25 (1.61) | .42 (3.13) |
| C | .336 (2.36) | .25 (1.61) | .42 (3.13) |
| D | .559 (4.44) | .44 (3.25) | .72 (6.41) |
| E | .564 (4.50) | .44 (3.25) | .72 (6.41) |
| F | .602 (4.93) | .44 (3.25) | .72 (6.41) |
| G | .622 (5.16) | .49 (3.73) | .78 (7.39) |
| H | .627 (5.21) | .49 (3.73) | .78 (7.39) |
| I | .658 (5.61) | .49 (3.73) | .78 (7.39) |
| J | .902(10.615) | .79 (7.59) | .97 (15.1) |
| K | .905 (10.72) | .79 (7.59) | .97 (15.1) |
| L | .924 (11.54) | .79 (7.59) | .97 (15.1) |

Table S4: Type I error rate for the MR-DoC model given the scenarios described in Table S1 and a *non-pleiotropic* instrumental variable (parameter b_2_ = 0). We simulated 10 000 samples under the null model of no causal effect of the exposure on the outcome variable (parameter g_1_ equalled 0). Each sample consisted of 2000 twin pairs. The type I error was calculated as the percentage of datasets in which the null hypothesis was incorrectly rejected given two significance thresholds, 0.05 and 0.01. In fitting the MR-DoC model we constrained re to equal 0 (as simulated). Abbreviation: CI – Confidence Interval.

| Scenario (S1) | alpha=0.01 [99% CI] | alpha=0.05 [99% CI] |
| --- | --- | --- |
| A | 0.0103 [0.0079, 0.0133] | 0.0505 [0.0451, 0.0564] |
| B | 0.0079 [0.0058, 0.0105] | 0.049 [0.0436, 0.0549] |
| C | 0.0096 [0.0073, 0.0125] | 0.0499 [0.0445, 0.0558] |
| D | 0.0111 [0.0086, 0.0141] | 0.0501 [0.0447, 0.0560] |
| E | 0.0106 [0.0082, 0.0136] | 0.0498 [0.0444, 0.0557] |
| F | 0.0107 [0.0083, 0.0137] | 0.0496 [0.0442, 0.0555] |
| G | 0.0106 [0.0082, 0.0136] | 0.0511 [0.0456, 0.0571] |
| H | 0.0114 [0.0089, 0.0145] | 0.0519 [0.0464, 0.0579] |
| I | 0.0102 [0.0078, 0.0131] | 0.0506 [0.0451, 0.0566] |
| J | 0.0106 [0.0082, 0.0136] | 0.0505 [0.0451, 0.0564] |
| K | 0.0095 [0.0072, 0.0124] | 0.0494 [0.0440, 0.0553] |
| L | 0.0094 [0.0071, 0.0122] | 0.0484 [0.0431, 0.0542] |

Table S5: Simulation results given a *non-pleiotropic* instrumental variable (b_2_=0). We report the power to detect the causal effect g_1_ (given alpha of 0.05) and the NCP obtained based on the standard MR with the causal effect estimated using two-stage least squares (N=2000 unrelateds), and based on the MR-DoC twin model (N=2000 twin pairs). In fitting the MR-DoC model we constrained re to equal 0 (as simulated).

| Scenario (S1) | Power (NCP) based on 2000 twin-pairs | Standard MR’s power (NCP) based on 2000 unrelateds  (twin 1) | Standard MR’s power (NCP)  in 4000 unrelateds |
| --- | --- | --- | --- |
| A | >.99 (38.68) | .25 (1.61) | .42 (3.13) |
| B | .984 (17.00) | .25 (1.61) | .42 (3.13) |
| C | .857 (9.18) | .25 (1.61) | .42 (3.13) |
| D | >.99 (40.52) | .44 (3.25) | .72 (6.41) |
| E | .992 (19.15) | .44 (3.25) | .72 (6.41) |
| F | .927 (11.66) | .44 (3.25) | .72 (6.41) |
| G | >.99 (88.54) | .45 (3.73) | .78 (7.39) |
| H | >.99 (40.24) | .45 (3.73) | .78 (7.39) |
| I | >.99 (21.42) | .45 (3.73) | .78 (7.39) |
| J | >.99 (91.83) | .79 (7.59) | .97 (15.1) |
| K | >.99 (44.83) | .79 (7.59) | .97 (15.1) |
| L | >.99 (26.93) | .79 (7.59) | .97 (15.1) |

Table S6: Scenarios and parameter values used to generate data for assessing the power of the MR–DoC twin model, given a *pleiotropic* instrumental variable and no unique environmental correlation (parameters b_2_≠0 and re=0). We varied the strength of the instrument, (i.e., the Polygenic Score explained either 0.05 or 0.1 proportion of the variance in the exposure), the proportion of variance explained in the exposure (X) and the outcome (Y) variable by additive genetic (h^2^_X_ =σ^2^_Ax_/σ^2^_phX_; h^2^_Y_ = σ^2^_Ay_/σ^2^_phY_), shared environmental (c^2^_X_ =σ^2^_Cx_/σ^2^_phX_; c^2^_Y_ = σ^2^_Cy_/σ^2^_phY_) and unique environmental (e^2^_X_ =σ^2^_Ex_/σ^2^_phX_; e^2^_Y_ = σ^2^_Ey_/σ^2^_phY_) factors, and the contribution of the instrumental variable (PGS; parameter b_1_), the causal effect (g_1_) and of the residual correlation between the outcome and the exposure (r_ξXξY_) to the 10% explained variance in the outcome. The effect size (see the five components of variance C1 to C5 in Fig.5) is defined as the percentage of explained variance in the outcome given the chosen parameter values b_1_, b_2_, g_1_, σ_ξXξY_ (covariance of ξX and ξY), σ^2^_ξX_ (residual variance in X), and σ^2^_ξY_ (residual variance in Y).

| Scenario (S2) | Parameter values | Effect size: percentage (%) of variance explained in the outcome (variance component in Fig. 5) |
| --- | --- | --- |
| A | PGS = 0.05  h^2^_X_ = 0.2, c^2^_X_ = 0.1, e^2^_X_ = 0.7  h^2^_Y_ = 0.5, c^2^_Y_ = 0.2, e^2^_Y_ = 0.3  r_ξXξY_ = 0.4 | b_1_^2^ × g_1_^2^ = 0.027 (C1)  2 × b_1_ × g_1_ × b_2_ = 0.607 (C2)  b_2_^2^ = 3.364 (C3)  g_1_^2^ × σ^2^_ξX_ = 0.521 (C4)  2 × g_1_ × σ_ξXξY_ = 5.478 (C5) |
| B | PGS = 0.05  h^2^_X_ = 0.5, c^2^_X_ = 0.2, e^2^_X_ = 0.3  h^2^_Y_ = 0.5, c^2^_Y_ = 0.2, e^2^_Y_ = 0.3  r_ξXξY_ = 0.4 | b_1_^2^ × g_1_^2^ = 0.027 (C1)  2 × b_1_ × g_1_ × b_2_ = 0.607 (C2)  b_2_^2^ = 3.364 (C3)  g_1_^2^ × σ^2^_ξX_ = 0.521 (C4)  2 × g_1_ × σ_ξXξY_ = 5.478 (C5) |
| C | PGS = 0.05  h^2^_X_ = 0.5, c^2^_X_ = 0.2, e^2^_X_ = 0.3  h^2^_Y_ = 0.2, c^2^_Y_ = 0.1, e^2^_Y_ = 0.7  r_ξXξY_ = 0.4 | b_1_^2^ × g_1_^2^ = 0.027 (C1)  2 × b_1_ × g_1_ × b_2_ = 0.607 (C2)  b_2_^2^ = 3.364 (C3)  g_1_^2^ × σ^2^_ξX_ = 0.521 (C4)  2 × g_1_ × σ_ξXξY_ = 5.478 (C5) |
| D | PGS = 0.1  h^2^_X_ = 0.2, c^2^_X_ = 0.1, e^2^_X_ = 0.7  h^2^_Y_ = 0.5, c^2^_Y_ = 0.2, e^2^_Y_ = 0.3  r_ξXξY_ = 0.4 | b_1_^2^ × g_1_^2^ = 0.057 (C1)  2 × b_1_ × g_1_ × b_2_ = 0.846 (C2)  b_2_^2^ = 3.095 (C3)  g_1_^2^ × σ^2^_ξX_ = 0.521 (C4)  2 × g_1_ × σ_ξXξY_ = 5.478 (C5) |
| E | PGS = 0.1  h^2^_X_ = 0.5, c^2^_X_ = 0.2, e^2^_X_ = 0.3  h^2^_Y_ = 0.5, c^2^_Y_ = 0.2, e^2^_Y_ = 0.3  r_ξXξY_ = 0.4 | b_1_^2^ × g_1_^2^ = 0.057 (C1)  2 × b_1_ × g_1_ × b_2_ = 0.846 (C2)  b_2_^2^ = 3.095 (C3)  g_1_^2^ × σ^2^_ξX_ = 0.521 (C4)  2 × g_1_ × σ_ξXξY_ = 5.478 (C5) |
| F | PGS = 0.1  h^2^_X_ = 0.5, c^2^_X_ = 0.2, e^2^_X_ = 0.3  h^2^_Y_ = 0.2, c^2^_Y_ = 0.1, e^2^_Y_ = 0.7  r_ξXξY_ = 0.4 | b_1_^2^ × g_1_^2^ = 0.057 (C1)  2 × b_1_ × g_1_ × b_2_ = 0.846 (C2)  b_2_^2^ = 3.095 (C3)  g_1_^2^ × σ^2^_ξX_ = 0.521 (C4)  2 × g_1_ × σ_ξXξY_ = 5.478 (C5) |
| G | PGS = 0.05  h^2^_X_ = 0.2, c^2^_X_ = 0.1, e^2^_X_ = 0.7  h^2^_Y_ = 0.5, c^2^_Y_ = 0.2, e^2^_Y_ = 0.3  r_ξXξY_ = 0.2 | b_1_^2^ × g_1_^2^= 0.075 (C1)  2 × b_1_ × g_1_ × b_2_ = 0.95 (C2)  b_2_^2^ =2.973 (C3)  g_1_^2^ × σ^2^_ξX_ = 1.442 (C4)  2 × g_1_ × σ_ξXξY_ = 4.557 (C5) |
| H | PGS = 0.05  h^2^_X_ = 0.5, c^2^_X_ = 0.2, e^2^_X_ = 0.3  h^2^_Y_ = 0.5, c^2^_Y_ = 0.2, e^2^_Y_ = 0.3  r_ξXξY_ = 0.2 | b_1_^2^ × g_1_^2^ = 0.075 (C1)  2 × b_1_ × g_1_ × b_2_ = 0.95 (C2)  b_2_^2^ =2.973 (C3)  g_1_^2^ × σ^2^_ξX_ = 1.442 (C4)  2 × g_1_ × σ_ξXξY_ = 4.557 (C5) |
| I | PGS = 0.05  h^2^_X_ = 0.5, c^2^_X_ = 0.2, e^2^_X_ = 0.3  h^2^_Y_ = 0.2, c^2^_Y_ = 0.1, e^2^_Y_ = 0.7  r_ξXξY_ = 0.2 | b_1_^2^ × g_1_^2^ = 0.075 (C1)  2 × b_1_ × g_1_ × b_2_ = 0.95 (C2)  b_2_^2^ =2.973 (C3)  g_1_^2^ × σ^2^_ξX_ = 1.442 (C4)  2 × g_1_ × σ_ξXξY_ = 4.557 (C5) |
| J | PGS = 0.1  h^2^_X_ = 0.2, c^2^_X_ = 0.1, e^2^_X_ = 0.7  h^2^_Y_ = 0.5, c^2^_Y_ = 0.2, e^2^_Y_ = 0.3  r_ξXξY_ = 0.2 | b_1_^2^ × g_1_^2^ = 0.16 (C1)  2 × b_1_ × g_1_ × b_2_ = 1.28 (C2)  b_2_^2^ =2.558 (C3)  g_1_^2^ × σ^2^_ξX_ =1.442 (C4)  2 × g_1_ × σ_ξXξY_ = 4.557 (C5) |
| K | PGS = 0.1  h^2^_X_ = 0.5, c^2^_X_ = 0.2, e^2^_X_ = 0.3  h^2^_Y_ = 0.5, c^2^_Y_ = 0.2, e^2^_Y_ = 0.3  r_ξXξY_ = 0.2 | b_1_^2^ × g_1_^2^ = 0.16 (C1)  2 × b_1_ × g_1_ × b_2_ = 1.28 (C2)  b_2_^2^ =2.558 (C3)  g_1_^2^ × σ^2^_ξX_ =1.442 (C4)  2 × g_1_ × σ_ξXξY_ = 4.557 (C5) |
| L | PGS = 0.1  h^2^_X_ = 0.5, c^2^_X_ = 0.2, e^2^_X_ = 0.3  h^2^_Y_ = 0.2, c^2^_Y_ = 0.1, e^2^_Y_ = 0.7  r_ξXξY_ = 0.2 | b_1_^2^ × g_1_^2^ = 0.16 (C1)  2 × b_1_ × g_1_ × b_2_ = 1.28 (C2)  b_2_^2^ =2.558 (C3)  g_1_^2^ × σ^2^_ξX_ =1.442 (C4)  2 × g_1_ × σ_ξXξY_ = 4.557 (C5) |

Table S7: Type I error rate for the MR-DoC model given the scenarios described in Table S6 (see above) and a *pleiotropic* instrumental variable (parameter b_2_≠0). We simulated 10 000 samples under the null model of no causal effect of the exposure on the outcome variable (parameter g_1_ equalled 0). Each sample consisted of 2000 twin pairs. The type I error was calculated as the percentage of datasets in which the null hypothesis was incorrectly rejected given two significance thresholds, 0.05 and 0.01. In fitting the MR-DoC model, to render the model identified, we assumed that parameter re equals 0 (as simulated). Abbreviation: CI – Confidence Interval.

| Scenario (S1) | alpha=0.01 [99% CI] | alpha=0.05 [99% CI] |  |
| --- | --- | --- | --- |
| A | 0.0098 [0.0075, 0.0127] | 0.0505 [0.0451, 0.0564] | 1 |
| B | 0.0095 [0.0072, 0.0124] | 0.046 [0.0408, 0.0517] | 2 |
| C | 0.01 [0.0077, 0.0129] | 0.0487 [0.0434, 0.0546] | 3 |
| D | 0.0094 [0.0071, 0.0122] | 0.0509 [0.0454, 0.0569] | 4 |
| E | 0.0095 [0.0072, 0.0124] | 0.0479 [0.0426, 0.0537] | 5 |
| F | 0.0104 [0.0080, 0.0134] | 0.0515 [0.0460, 0.0575] | 6 |
| G | 0.0095 [0.0072, 0.0124] | 0.0496 [0.0442, 0.0555] | 7 |
| H | 0.0109 [0.0084, 0.0139] | 0.0481 [0.0428, 0.0539] | 8 |
| I | 0.0104 [0.0080, 0.0134] | 0.0482 [0.0429, 0.0540] | 9 |
| J | 0.0116 [0.0091, 0.0147] | 0.0512 [0.0457, 0.0572] | 10 |
| K | 0.0099 [0.0076, 0.0128] | 0.0489 [0.0435, 0.0548] | 11 |
| L | 0.0104 [0.0080, 0.0134] | 0.0483 [0.0430, 0.0541] | 12 |

Table S8: Simulation results given a *pleiotropic* instrumental variable (parameter b_2_≠0) and the scenarios described in Table S6 (above). We report the power to detect the causal effect g_1_ (given alpha=0.05), and the NCP based on the MR-DoC twin model (N=2000 twin pairs). In fitting the MR-DoC model, to render the model identified, we assumed that parameter re equals 0 (as simulated).

| Scenario (S2) | Power (NCP) |
| --- | --- |
| A | .97 (14.77) |
| B | .686 (5.98) |
| C | .379 (2.73) |
| D | .970 (14.77) |
| E | .686 (5.98) |
| F | .379 (2.73) |
| G | >.99 (39.1) |
| H | .981 (16.28) |
| I | .770 (7.29) |
| J | >.99 (39.1) |
| K | .981 (16.28) |
| L | .77 (7.29) |

Table S9: Scenarios and parameter values used to generate data for calculating the number of twin pairs needed by MR–DoC twin model to achieve various power levels, given a *pleiotropic* instrumental variable (parameter b_2_≠0), an ACE trait as the exposure and an AE trait as the outcome. The Polygenic Score explained 0.05 proportion of the variance in the exposure). We varied the proportion of variance explained in the exposure (X) and the outcome (Y) variable by additive genetic (h^2^_X_=σ^2^_Ax_/σ^2^_phX_; h^2^_Y_=σ^2^_Ay_/σ^2^_phY_), shared environmental (c^2^_X_=σ^2^_Cx_/σ^2^_phX_) and unique environmental (e^2^_X_=σ^2^_Ex_/σ^2^_phX_; e^2^_Y_=σ^2^_Ey_/σ^2^_phY_) factors, and the contribution of the instrumental variable (PGS; parameter b_1_), the causal effect (g_1_) and of the residual correlation between the outcome and the exposure (r_ξXξY_) to the 10% explained variance in the outcome. The effect size (see the five components of variance C1 to C5 in Fig.5) is defined as the percentage of explained variance in the outcome given the chosen parameter values b_1_, b_2_, g_1_, σ_ξXξY_ (covariance of ξX and ξY), σ^2^_ξX_ (residual variance in X), and σ^2^_ξY_ (residual variance in Y). With an ACE trait as the exposure and an AE trait as an outcome we estimate re, b_2_ as well as g_1._

| Scenario (S3) | Parameter values | Effect size: percentage (%) of variance explained in the outcome (variance component in Fig. 5) |
| --- | --- | --- |
| A | h^2^_X_ = 0.2, c^2^_X_ = 0.3, e^2^_X_ = 0.5  h^2^_Y_ = 0.1, e^2^_Y_ = 0.9  r_ξXξY_ = 0.4 | b_1_^2^ × g_1_^2^ = 0.027 (C1)  2 × b_1_ × g_1_ × b_2_ = 0.607 (C2)  b_2_^2^ = 3.364 (C3)  g_1_^2^ × σ^2^_ξX_ = 0.521 (C4)  2 × g_1_ × σ_ξXξY_ =5.478 (C5) |
| B | h^2^_X_ = 0.2, c^2^_X_ = 0.3, e^2^_X_ = 0.5  h^2^_Y_ = 0.6, e^2^_Y_ = 0.4  r_ξXξY_ = 0.4 | b_1_^2^ × g_1_^2^ = 0.027 (C1)  2 × b_1_ × g_1_ × b_2_ = 0.607 (C2)  b_2_^2^ = 3.364 (C3)  g_1_^2^ × σ^2^_ξX_ = 0.521 (C4)  2 × g_1_ × σ_ξXξY_ =5.478 (C5) |
| C | h^2^_X_ = 0.2, c^2^_X_ = 0.2, e^2^_X_ = 0.6  h^2^_Y_ = 0.1, e^2^_Y_ = 0.9  r_ξXξY_ = 0.4 | b_1_^2^ × g_1_^2^ = 0.027 (C1)  2 × b_1_ × g_1_ × b_2_ = 0.607 (C2)  b_2_^2^ = 3.364 (C3)  g_1_^2^ × σ^2^_ξX_ = 0.521 (C4)  2 × g_1_ × σ_ξXξY_ =5.478 (C5) |
| D | h^2^_X_ = 0.2, c^2^_X_ = 0.2, e^2^_X_ = 0.6  h^2^_Y_ = 0.6, e^2^_Y_ = 0.4  r_ξXξY_ = 0.4 | b_1_^2^ × g_1_^2^ = 0.027 (C1)  2 × b_1_ × g_1_ × b_2_ = 0.607 (C2)  b_2_^2^ = 3.364 (C3)  g_1_^2^ × σ^2^_ξX_ = 0.521 (C4)  2 × g_1_ × σ_ξXξY_ =5.478 (C5) |
| E | h^2^_X_ = 0.2, c^2^_X_ = 0.3, e^2^_X_ = 0.5  h^2^_Y_ = 0.1, e^2^_Y_ = 0.9  r_ξXξY_ = 0.2 | b_1_^2^ × g_1_^2^ = 0.075 (C1)  2 × b_1_ × g_1_ × b_2_ = 0.95 (C2)  b_2_^2^ = 2.973 (C3)  g_1_^2^ × σ^2^_ξX_ = 1.442 (C4)  2 × g_1_ × σ_ξXξY_ = 4.557 (C5) |
| F | h^2^_X_ = 0.2, c^2^_X_ = 0.3, e^2^_X_ = 0.5  h^2^_Y_ = 0.6, e^2^_Y_ = 0.4  r_ξXξY_ = 0.2 | b_1_^2^ × g_1_^2^ = 0.075 (C1)  2 × b_1_ × g_1_ × b_2_ = 0.95 (C2)  b_2_^2^ = 2.973 (C3)  g_1_^2^ × σ^2^_ξX_ = 1.442 (C4)  2 × g_1_ × σ_ξXξY_ = 4.557 (C5) |
| G | h^2^_X_ = 0.2, c^2^_X_ = 0.2, e^2^_X_ = 0.6  h^2^_Y_ = 0.1, e^2^_Y_ = 0.9  r_ξXξY_ = 0.2 | b_1_^2^ × g_1_^2^ = 0.075 (C1)  2 × b_1_ × g_1_ × b_2_ = 0.95 (C2)  b_2_^2^ = 2.973 (C3)  g_1_^2^ × σ^2^_ξX_ = 1.442 (C4)  2 × g_1_ × σ_ξXξY_ = 4.557 (C5) |
| H | h^2^_X_ = 0.2, c^2^_X_ = 0.2, e^2^_X_ = 0.6  h^2^_Y_ = 0.6, e^2^_Y_ = 0.4  r_ξXξY_ = 0.2 | b_1_^2^ × g_1_^2^ = 0.075 (C1)  2 × b_1_ × g_1_ × b_2_ = 0.95 (C2)  b_2_^2^ = 2.973 (C3)  g_1_^2^ × σ^2^_ξX_ = 1.442 (C4)  2 × g_1_ × σ_ξXξY_ = 4.557 (C5) |

Table S10: Type I error rate for the MR-DoC model given the scenarios described in Table S9 (see above). We simulated 1000 samples under the null model of no causal effect of the exposure on the outcome variable (parameter g_1_ equalled 0). Each sample consisted of 20K twin pairs. The type I error was calculated as the percentage of datasets in which the null hypothesis was incorrectly rejected given two significance thresholds, 0.05 and 0.01. With an ACE trait as the exposure and an AE trait as an outcome we estimate re, b_2_ as well as g_1_ (simulated g_1_=0)_._ Abbreviation: CI – Confidence Interval.

| Scenario (S3) | alpha=0.01 [99% CI] | alpha=0.05 [99% CI] |
| --- | --- | --- |
| A | 0.008[0.0030, 0.0198] | 0.045[0.0304,0.0656] |
| B | 0.008[0.0030, 0.0198] | 0.043[0.0288,0.0633] |
| C | 0.008[0.0030, 0.0198] | 0.048[0.0329,0.0691] |
| D | 0.01[0.0045,0.0219] | 0.049[0.0337,0.0703] |
| E | 0.011 [0.0048,0.0239] | 0.052[0.0362,0.0737] |
| F | 0.008[0.0030, 0.0198] | 0.042[0.0280,0.0621] |
| G | 0.007[0.0024,0.0184] | 0.042[0.0280,0.0621] |
| H | 0.008[0.0030, 0.0198] | 0.044[0.0296,0.0645] |

Figure S1: Number of twin pairs needed by MR–DoC twin model to achieve various power levels, given a *pleiotropic* instrumental variable (parameter b_2_≠0), an ACE trait as the exposure and an AE trait as the outcome (see Table S9 above for details on scenarios and parameter values used to generate data). Power increases with increasing proportion of variance explained by shared environmental factors in the exposure (c^2^_X_), with decreasing residual correlation between the outcome and the exposure (r_ξXξY_), and with increasing heritability of the outcome variable (h^2^_Y_). Abbreviations: K = 1000.


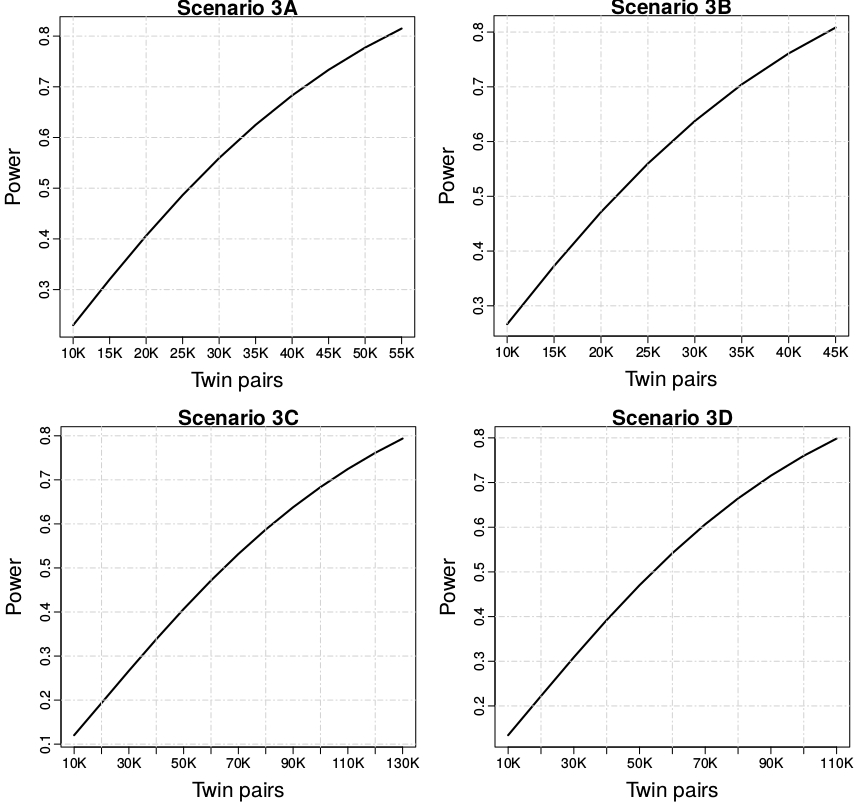


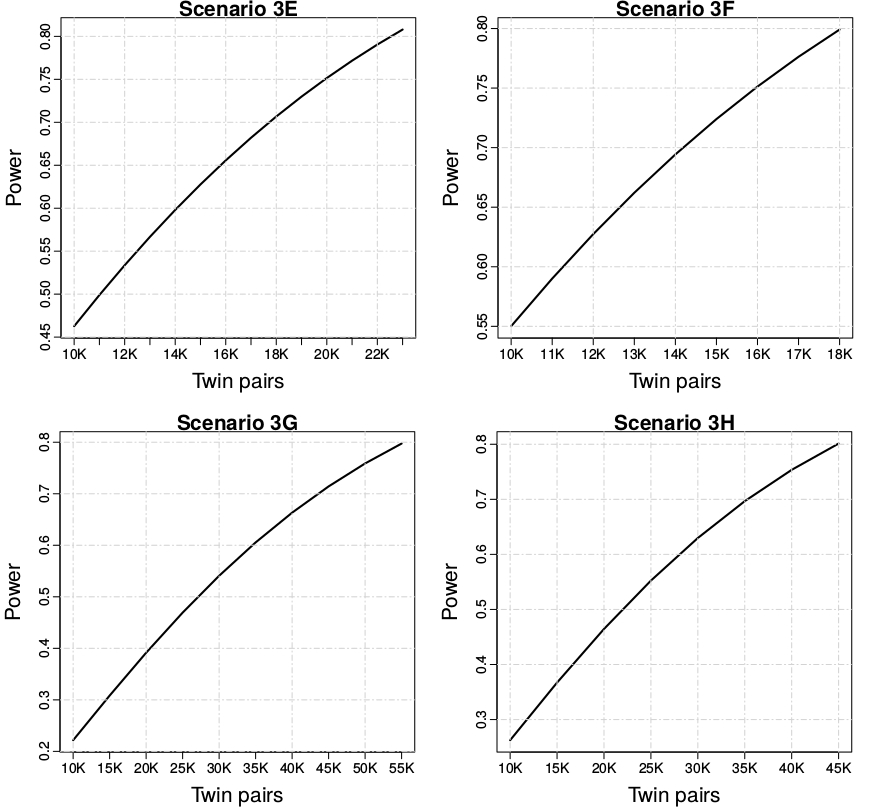

Supplement: Supplementary file 1 — Supplementary material 1 (DOCX 446 KB) [file 10519_2018_9904_MOESM1_ESM.docx]
